# Chromatin remodeling in *Drosophila* preblastodermic embryo extract

**DOI:** 10.1038/s41598-018-29129-8

**Published:** 2018-07-19

**Authors:** Eva Šatović, Jofre Font-Mateu, Albert Carbonell, Miguel Beato, Fernando Azorín

**Affiliations:** 1 Institute of Molecular Biology of Barcelona, CSIC, Barcelona, Spain; 2 Institute for Research in Biomedicine, IRB Barcelona. The Barcelona Institute for Science and Technology, Barcelona, Spain; 3 Ruder Boskovic Institute, Zagreb, Croatia; 4 Centre de Regulació Genòmica (CRG). The Barcelona Institute for Science and Technology, Barcelona, Spain; 5 Universitat Pompeu Fabra (UPF), Barcelona, Spain

## Abstract

Chromatin is known to undergo extensive remodeling during nuclear reprogramming. However, the factors and mechanisms involved in this remodeling are still poorly understood and current experimental approaches to study it are not best suited for molecular and genetic analyses. Here we report on the use of *Drosophila* preblastodermic embryo extracts (DREX) in chromatin remodeling experiments. Our results show that incubation of somatic nuclei in DREX induces changes in chromatin organization similar to those associated with nuclear reprogramming, such as rapid binding of the germline specific linker histone dBigH1 variant to somatic chromatin, heterochromatin reorganization, changes in the epigenetic state of chromatin, and nuclear lamin disassembly. These results raise the possibility of using the powerful tools of *Drosophila* genetics for the analysis of chromatin changes associated with this essential process.

## Introduction

Chromatin remodeling is essential for nuclear reprogramming. Experimental approaches to study chromatin remodeling during nuclear reprogramming include ectopic expression of Yamanaka transcription factors, nuclear transfer to eggs or oocytes, cell fusion with embryonic stem cells and *in vitro* treatment with oocyte or egg extracts^1–9^. Here we address the potential use of cell-free extracts prepared from *Drosophila* preblastoderm embryos (DREX) to study chromatin remodeling. DREX has been extensively used in exploring DNA replication^10^, chromatin assembly^11,12^ and decondensation^13,14^, nuclear formation^15^, nuclear envelope assembly^16,17^, and for the study of mitosis *ex vivo*^18^. We show that, upon incubation in DREX, somatic nuclei undergo important structural changes reminiscent of nuclear reprogramming. One of the earliest events during nuclear reprogramming is the exchange of somatic linker histones H1 by oocyte specific variants^7,19–22^. Metazoans usually contain multiple somatic^23,24^ and germline specific H1 variants^25^. However, in *Drosophila*, histone H1 complexity is reduced to a single somatic dH1 variant^26–28^, and a second germline specific dBigH1 isoform, which is also present in the early embryo until activation of the zygotic genome (ZGA) at cellularization^29^. Our results show that incubation of somatic nuclei in DREX results in rapid binding of dBigH1. In addition, we also show that DREX induces heterochromatin reorganization, nuclear lamin disassembly and changes in the pattern of histone modifications, all of which are associated with reprogramming of somatic nuclei. These results suggest that DREX induces partial remodeling of somatic chromatin, opening up the possibility of using the powerful tools of *Drosophila* genetics to study this central step in somatic cells reprogramming.

## Results

### Incubation of somatic nuclei in DREX induces dBigH1 incorporation

**Figure 1:**
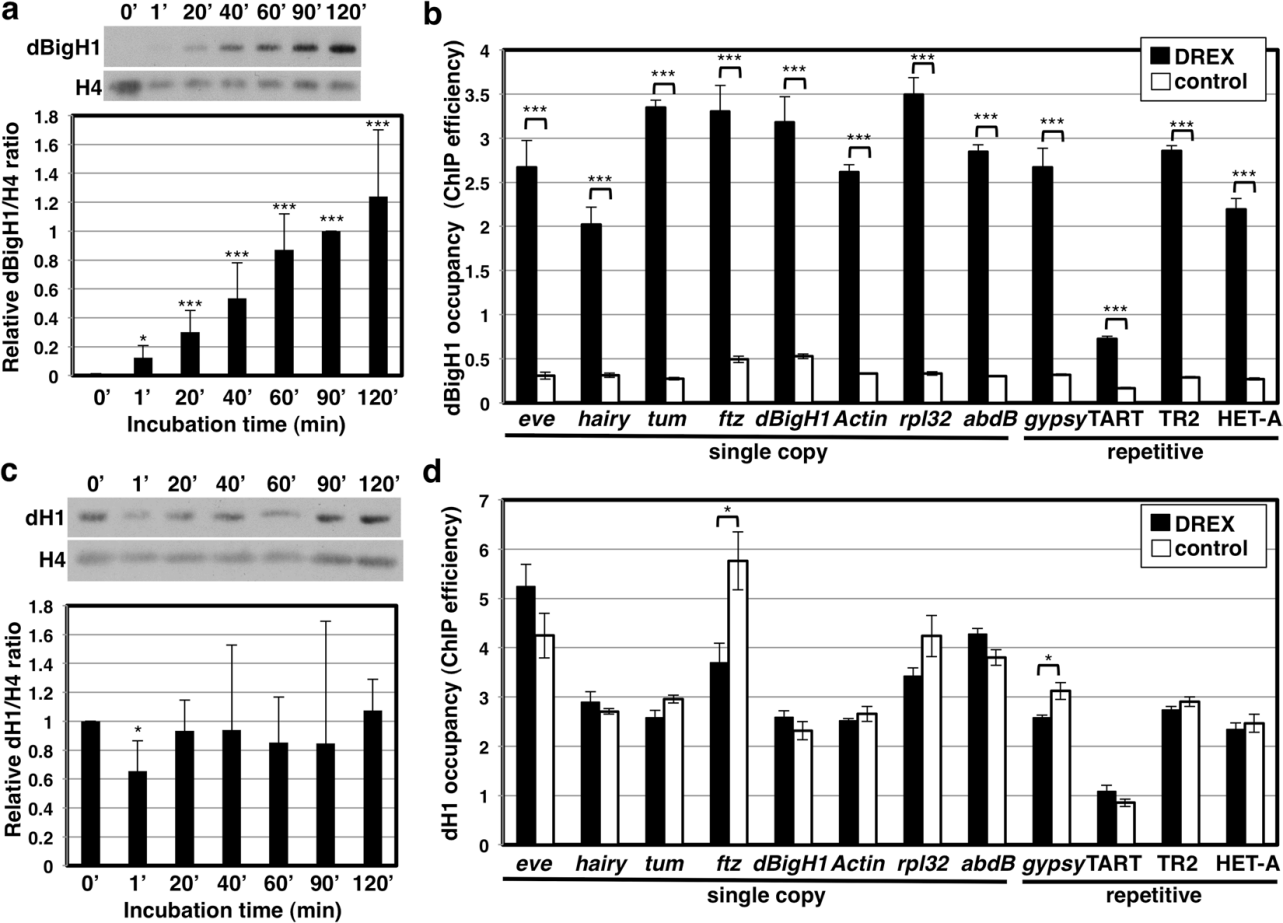
Incubation in DREX induces binding of dBigH1 to somatic S2 nuclei chromatin. (**a**) Western blot (WB) analysis of the amount of dBigH1 bound to S2 chromatin after incubation in DREX for the indicated times. Quantitative analysis of the results is shown in the bottom. Data are presented as mean ± SD. (N = 3; two-tailed T-test p-values: *< 0.05, ***< 0.001). (**b**) dBigH1 ChIP-qPCR analysis at the indicated genomic elements after incubation of somatic S2 nuclei for 1 h in DREX or in control conditions. Results are presented as % of input. Data are presented as normalized mean ± SEM (N = 3; two-tailed T-test p-values: ***< 0.001). (**c**) As in (**a**) but for αdH1. Data are presented as mean ± SD. (N = 3; two-tailed T-test p-values: *< 0.05) (**d**) As in (**b**) but for dH1. Data are presented as mean ± SEM (N = 3; two-tailed T-test p-values: *< 0.05). Full-size films of the blots are presented in Supplementary Figures S6 and S7 (biological replicates).

An early event in reprogramming of somatic nuclei transplanted into oocytes is the binding to chromatin of the oocyte specific linker histones H1^7,19–22^. In this regard, incubation of somatic nuclei prepared from *Drosophila* S2 cells in DREX, which is enriched in dBigH1, resulted in its incorporation to chromatin. Immunofluorescence analyses (IF) showed a clear association of dBigH1 with S2 nuclei after incubation in DREX (Fig. S1a). Notice that no dBigH1 was detected in S2 nuclei prior to incubation. Furthermore, fractionation into soluble nuclear and chromatin bound material, detected the presence of dBigH1 in the chromatin bound fraction (Fig. 1a). dBigH1 binding was detected as early as 1′ after incubation in DREX and increased progressively during incubation (Fig. 1a). ChIP-qPCR analysis confirmed these results since, after incubation in DREX, significant dBigH1 occupancy was detected at multiple genomic sites, both single-copy and repetitive (Fig. 1b), suggesting that dBigH1 binding occurred across chromatin.

In nuclear transfer (NT) experiments, binding of the oocyte specific H1s usually results in displacement of the corresponding somatic H1s^8,19–21,30,31^. In this regard, no significant reduction in the amount of chromatin bound dH1 was detected after incubation in DREX for up to 2 h (Fig. 1c). In addition, ChIP-qPCR experiments showed that dH1 occupancy was not impaired upon dBigH1 binding (Fig. 1d). Furthermore, IF experiments detected only a weak negative correlation between dBigH1 and dH1 content in DREX-treated nuclei (Fig. S1b). Altogether, these results suggest that dBigH1 binding occurs without significant dH1 displacement. Notice, however, that after 1′ of incubation dH1 content significantly decreased though dBigH1 binding was only weak (Fig. 1c).

### Incubation in DREX induces histone acetylation

**Figure 2:**
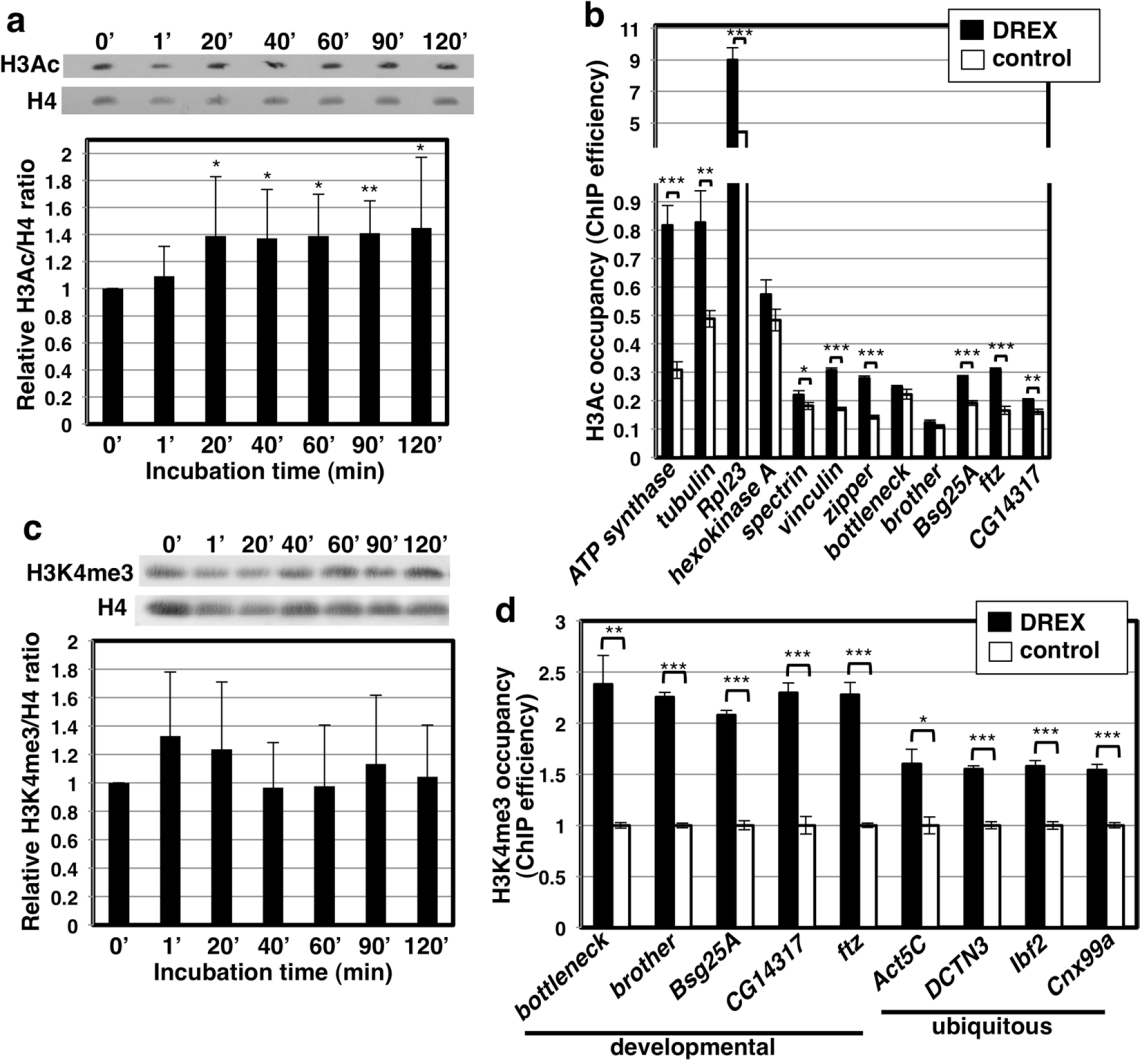
Incubation in DREX alters the epigenetic state of chromatin. (**a**) WB analyses of the global levels of H3Ac after incubation in DREX for the indicated times. Data are presented as normalized mean ± SD (N = 3; two-tailed T-test p-values: *<0.05, **<0.01) (**b**) H3Ac ChIP-qPCR analysis at the indicated genes after incubation of somatic S2 nuclei in DREX or in control conditions for 1 h. Results are presented as % of input. Data are presented as normalized mean ± SEM (N = 3; two-tailed T-test p-values: *<0.5, **<0–01, ***<0.001). (**c**) As in (**a**) but for global levels of H3K4me3. Data are presented as mean ± SD (N = 3; two-tailed T-test p-values > 0.05). (**d**) H3K4me3 ChIP-qPCR analysis at the promoters of the indicated genes after incubation of somatic S2 nuclei for 1 h in DREX. Results are presented as % of input. Data are presented as normalized mean ± SEM (N = 3; two-tailed T-test p-values: *<0.05, **<0.01, ***<0.001). Full-size films or scans of the blots are presented in Supplementary Figures S8 and S9 (biological replicates).

Nuclear reprogramming is usually accompanied by changes in the epigenetic state of chromatin^32–35^. In this regard, we observed that incubation in DREX resulted in a significant increase of global H3Ac (Fig. 2a). ChIP-qPCR experiments confirmed that H3Ac levels increased at multiple loci after incubation in DREX for 2 h (Fig. 2b). Increased H3Ac observed upon incubation in DREX was not associated with dBigH1 binding since a similar increase was also observed when dBigH1 binding was strongly impaired by the addition of αdBigH1 antibodies to DREX (Fig. S2). Increased histone acetylation suggests that incubation in DREX induces transition to a more active chromatin conformation. Thus, we also analyzed whether incubation in DREX affected the levels of H3K4me3, a modification accumulating at promoters of active genes. In this regard, although incubation in DREX did not significantly affect global H3K4me3 levels (Fig. 2c), we detected increased H3K4me3 levels at promoters of several genes (Fig. 2d). This increase was higher at promoters of developmentally regulated genes, highly expressed during early embryogenesis but silent in S2 cells, than at ubiquitously expressed genes (Fig. 2d). We noticed that global H3K4me3 showed a tendency to increase at short incubation times (p-value = 0.1309 at 1′) (Fig. 2c). Similarly, although global levels of the chromatin bound active RNApol II forms were not significantly affected (Fig. S3a and S3b), they showed a tendency to increase at short incubation times, in particular those of the promoter-proximal IIo^ser5^ form (Fig. S3a) (p-value = 0.1779 at 20′). H3K27me3 also showed a tendency to increase (Fig. S3c). Altogether, these results suggest that incubation in DREX alters the epigenetic landscape of somatic chromatin.

### DREX induces heterochromatin reorganization and nuclear lamin disassembly

**Figure 3:**
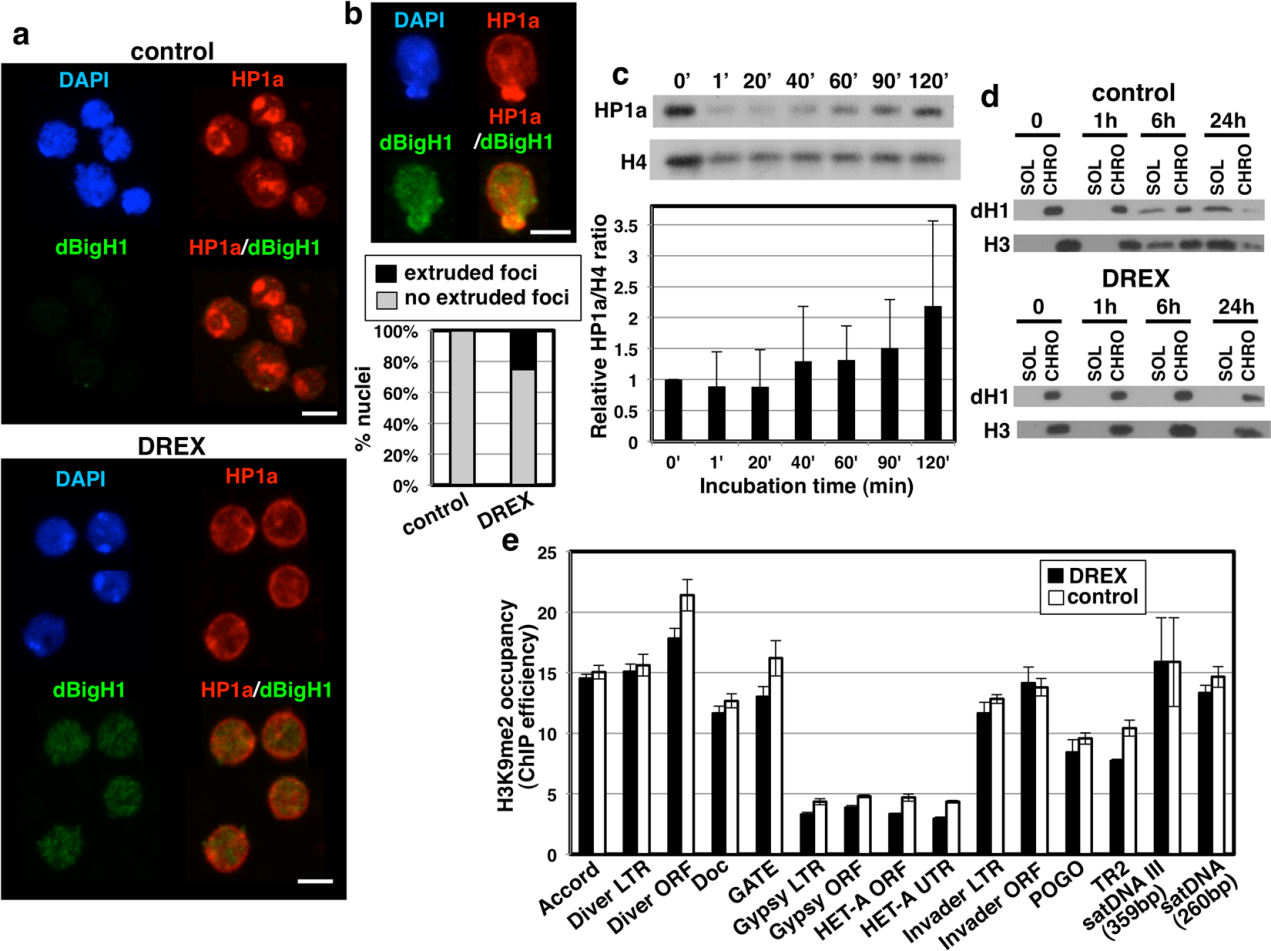
Incubation in DREX induces heterochromatin reorganization. (**a**) IF with αHP1a (red) and αdBigH1 (green) of somatic S2 nuclei after incubation for 1 h in DREX (bottom) or in control (top) conditions. DNA was stained with DAPI (blue). Scale bars are 3 µm. (**b**) As in (**a**), but for nuclei showing extruded HP1a foci. In the bottom, the proportions of nuclei showing extruded HP1a foci or not are presented after incubation in DREX or in control conditions for 2 h. (N = 497 for DREX; N = 536 for control). (**c**) WB analyses of the global levels of HP1a after incubation in DREX for the indicated times. Data are presented as normalized mean ± SD (N = 2, two-tailed T-test p-values > 0.05). (**d**) WB analyses of the amounts of dH1 and H3 detected in the soluble (SOL) and chromatin-bound (CHRO) fractions after fractionation of S2 nuclei incubated in DREX (bottom) and in control conditions (top) for the indicated times. (**e**) H3K9me2 ChIP-qPCR analysis at the indicated repetitive heterochromatic elements after incubation of somatic S2 nuclei for 1 h in DREX or in control conditions. Results are presented as % of input. Data are presented as mean ± SEM (N = 3; two-tailed T-test p-values > 0.05). Full-size films or scans of the blots are presented in Supplementary Figures S10 and S11 (biological replicate).

Next, we analyzed whether incubation in DREX affected heterochromatin organization. For this purpose, we performed IF experiments using αHP1a antibodies, which mark heterochromatin. In somatic S2 cells, HP1a is distributed in distinct heterochromatic foci (Fig. 3a, top and Fig. S4a). Upon incubation in DREX, the αHP1a immunostaining pattern was altered, showing a more uniform staining along the nuclei with slight accumulation on the periphery (Fig. 3a, bottom and Fig. S4b). After 2 h of incubation, only ~15% of nuclei preserve the normal HP1a foci pattern. Loss of HP1a foci was not the consequence of a global reduction in HP1a content since global HP1a levels were not significantly reduced upon incubation in DREX (Fig. 3c), but, on the contrary, they tend to increase at long incubation times (p-value = 0.0879 at 2 h). We also observed that incubation in DREX induced the formation of aberrant HP1a foci extruding from the nuclear surface in ~30% of nuclei (Fig. 3b). Similar results were obtained when immunostaining was performed with αH3K9me3 antibodies, which also mark heterochromatin (Fig. S5). Also in this case, a uniform αH3K9me3 pattern was observed upon incubation in DREX (Fig. S5a) and ~30% of nuclei showed extruded H3K9me3 foci (Fig. S5b). It has been reported that somatic nuclei incubated in *Xenopus* egg extracts eventually disassemble and undergo apoptosis^36^. However, the major heterochromatin reorganization observed upon incubation in DREX did not reflect general chromatin destabilization and/or apoptosis since release of histones H3 and dH1 was not detected upon incubation in DREX for as long as 24 h (Fig. 3d, bottom). In contrast, chromatin disassembly was observed in control nuclei after 6 h of incubation in the absence of DREX (Fig. 3d, top). Furthermore, ChIP-qPCR experiments showed that incubation in DREX did not significantly reduce H3K9me2 occupancy at several heterochromatic elements, including different types of transposable elements (TE) and satellite DNAs (Fig. 3e), suggesting that heterochromatin stability was not significantly compromised.

**Figure 4:**
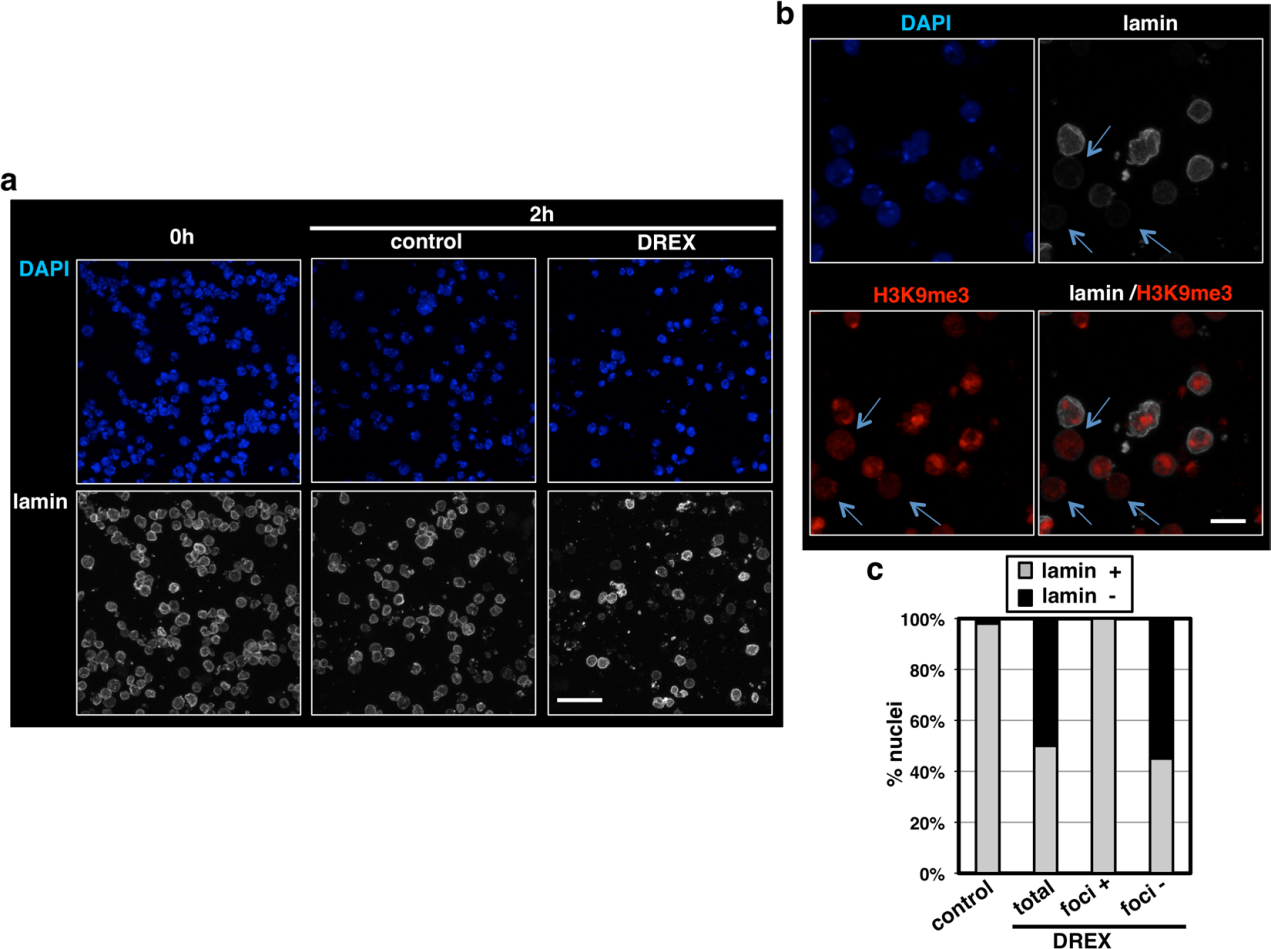
Incubation in DREX induces nuclear lamin disassembly. (**a**) IF with αlamin (white) of somatic S2 nuclei after incubation in DREX or in control conditions for 2 hours, and prior to incubation (0 h). DNA was stained with DAPI (blue). Scale bars are 20 µm. (**b**) As in (**a**), but staining also with αH3K9me3 (red). Arrows indicate nuclei showing diffuse αH3K9me3 staining. Scale bars are 7 µm. (**c**) The proportions of nuclei positive for αlamin or not are presented after incubation in DREX or in control conditions for 2 hours. After incubation in DREX, the proportions of αlamin positive/negative nuclei are also presented for nuclei showing H3K9me3 foci or not. (N = 202 for DREX; N = 184 for control).

Incubation in DREX also induced nuclear lamin disassembly as judged by IF (Fig. 4a). After 2 h of incubation, ~50% of nuclei showed no detectable αlamin reactivity at the nuclear envelope (NE), compared to only 2% in control nuclei (Fig. 4c). Interestingly, αlamin reactivity was generally not detected in nuclei showing diffuse αH3K9me3 immunostaining, while it was intense in nuclei with preserved αH3K9me3 foci (Fig. 4b). After 2 h of incubation in DREX, all nuclei with αH3K9me3 foci showed αlamin reactivity, while this proportion was reduced to ~45% in nuclei lacking usual αH3K9me3 foci (Fig. 4c). Altogether, these results suggest a correlation between heterochromatin reorganization and nuclear lamin disassembly.

## Discussion

Here we report that incubation of somatic nuclei in DREX induces changes in chromatin organization similar to those associated with nuclear reprogramming. On one hand, we observed rapid incorporation of the *Drosophila* germline specific linker histone dBigH1 into the somatic nuclei. NT experiments performed in *Xenopus* and mammals showed that incorporation of the oocyte specific linker histone variants B4 and H1oo into the donor nuclei is an early event in nuclear reprogramming^8,19–22,30,31^. B4 binding precedes loading of oocyte RNApol II and expression of a dominant negative B4 form significantly inhibits transcription of many reprogrammed genes^19^. Along the same lines, expression of H1oo in mouse ESCs impairs differentiation^37^ although it does not improve iPSC formation^38^. How oocyte specific H1s might contribute to nuclear reprogramming remains not well understood. Oocyte specific H1s are less positively charged than their somatic counterparts and, therefore, their interaction with DNA is weaker and condense chromatin less than somatic H1s^25^, rendering it more accessible to chromatin modifiers, remodelers and transcription factors^8,19,39^. In this regard, *Xenopus* B4 is more mobile than somatic H1^8^ and B4-containing chromatin is more accessible to remodeling factors^39^. B4 binds pervasively across chromatin of the donor nuclei and, concomitantly, somatic H1s are released^8,19^, suggesting competition of somatic H1s by the oocyte specific variants. However, this competition does not appear to play an important role in reprogramming since overexpression of somatic H1s does not interfere with B4 binding and subsequent activation of pluripotency genes^8^. Moreover, in mouse fibroblasts, binding of H1oo is detected 10′ after NT, while release of somatic H1s occurs later at 30′ after NT^20^. Similarly, somatic H1s replacement can last hours in NT experiments with bovine cells^21^. Finally, our results indicate that, upon incubation in DREX, dBigH1 binds along chromatin without affecting somatic dH1 occupancy. In fact, dH1 occupancy is significantly reduced only at short incubation times when dBigH1 binding is very low.

Our results also show that DREX induces changes in the epigenetic landscape of chromatin, which are in agreement with the global epigenetic remodeling of chromatin observed during reprogramming of somatic cells to iPSCs^32–35^. In particular, we observed increased global H3Ac that is maintained throughout the incubation time course. Increased histone acetylation is observed in fully reprogrammed iPSCs^40^ and ESC chromatin is hyperacetylated compared to differentiated cells^41–45^. We also observed that H3K4me3 levels increased more intensively at promoters of developmentally regulated genes that are silent in S2 cells but highly expressed in early embryogenesis, suggesting their reactivation. Interestingly, pluripotency-related and developmentally regulated genes are known to acquire H3K4me3 at promoters during nuclear reprogramming^32–35^. Finally, though not statistically significant, global levels of H3K4me3 and the chromatin bound promoter-proximal active RNApol IIo^ser5^ form tend to increase at short incubation times. In this regard, NT experiments in *Xenopus* showed loading of oocyte basal transcription factors and RNApol II leading to genome-wide transcriptional reprogramming and selective activation of pluripotency genes^19,46^. Notably, our results showed that increased histone acetylation induced by DREX does not require binding of dBigH1, suggesting that, at least in part, the epigenetic changes occurring during reprogramming do not depend only on the activities of the oocyte specific H1s.

Incubation in DREX also induces profound changes in chromatin/nuclear organization. On one hand, at short incubation times, DREX induces heterochromatin reorganization since HP1a/H3K9me3 foci disassemble. A decrease in the number of HP1a foci has also been reported during reprogramming to iPSC^40^. In this regard, chromatin of pluripotent cells is largely decondensed and heterochromatin is organized in larger and fewer domains that become smaller, more abundant and hypercondensed as cells differentiate^41,47–50^. Interestingly, incubation in DREX did not decrease H3K9me2 occupancy at multiple heterochromatic elements, suggesting that DREX affects condensation but not the actual heterochromatin content of somatic nuclei. Oocyte specific H1s might be one of the factors contributing to heterochromatin decondensation since, in humans, H1oo is required for decondensation of sperm chromatin^51^. At long incubation times, HP1a foci reform and extrude from nuclei. Interestingly, extrusion of heterochromatic sequences was also reported in somatic plant cells undergoing meiosis^52^. Finally, we also observed that DREX induces disassembly of nuclear lamin, a nuclear envelope component of differentiated cells that is absent in ESCs^53^. Similar results were reported earlier using a *Drosophila* oocyte cell-free extract^54^. Nuclear lamin disassembly is considered a marker of reprogrammed cells, since it is detected at the nuclear envelope in partial iPSCs, but not in fully reprogrammed iPSCs^40^. Interestingly, nuclear lamin disassembly strongly correlates with heterochromatin reorganization, which might account for the heterochromatin extrusion observed after long-term exposure to DREX.

In summary, our results show that DREX induces several changes associates with gain of pluripotency, such as binding of the germline specific linker histone dBigH1, epigenetic remodeling, heterochromatin reorganization and nuclear lamin disassembly. However, it is highly unlikely that DREX induces full reprogramming of somatic nuclei. Nevertheless, the use of DREX offers the possibility of applying the powerful genetics techniques developed in *Drosophila* to the analysis of factors and mechanisms involved in chromatin remodeling during this essential process.

## Materials and Methods

### Antibodies

Rabbit αdBigH1 antibodies are described in^29^ (1:5000 (WB), 1:4000 (IF)). Rabbit αdH1 antibodies were kindly provided by Dr. J. Kadonaga and are described in^55^ (1:20000 (WB), 1:4000 (IF)). Rat αHP1a antibodies are described in^56^ (1:10000 (WB), 1:400 (IF)). The rest of antibodies were commercially available: rabbit αH4 (Abcam ab10158, 1:15000 (WB)), rabbit αH3K4me3 (Abcam ab8580, 1:2000 (WB)), rabbit αH3Ac (Mill 06-599, 1:20000 (WB)), mouse αH3K9me2 (Abcam ab1220, ChIP 2 μl), rabbit αH3K9me3 (Mill 07-442, 1:75 (IF)), rabbit αPol II Ser2P (Abcam ab5095, 1:5000 (WB)), rabbit αPol II Ser5P (Abcam ab5131, 1:5000 (WB)), and mouse αlamin (DSHB ADL67.10, 1:1000 (WB)). Jackson and ThermoFisher commercial secondary antibodies were used for immunofluorescence, while IRDye 680LT and 800CW conjugates (LI-COR) and horseradish peroxidase-coupled antibody (Jackson) were used for WB detection.

### Incubation in DREX and cellular fractionation

DREX was prepared in Exb50 buffer (10 mM HEPES pH 7.6, 50 mM KCl, 1.5 mM MgCl_2_, 0.5 mM EGTA pH 8, 10 mM β-glicerophosphate, 10% Glycerol), as described in^57^. S2 cells were maintained in Schneider medium at 25 °C. Nuclei were isolated using Dounce homogenizer and Buffer A containing: 0.23 M sucrose, 15 mM Tris pH 7.4, 60 mM KCl, 0.25 mM MgCl_2_, 15 mM NaCl, 0.15 M spermine, 0.5 M spermidine, 0.2 mM PMSF, 14 mM β-MetOH. Purified nuclei were incubated in DREX for the indicated times. In control experiments, nuclei were incubated under the same experimental conditions in Exb50 buffer. After incubation nuclear fractionation was performed. Nuclear pellet was washed in Exb50 buffer and then lysed for 30 min in 10 mM HEPES pH 7.9, 3 mM EDTA, 0.2 mM EGTA, 1 mM DTT, Protease Inhibitor Cocktail. Centrifugation was carried out at 3000 g for 5 min resulting in supernatant (soluble nuclear fraction) and in pellet (chromatin).

### Immunostaining experiments

For immunostaining experiments nuclei incubated in DREX were washed, resuspended in PBS and placed on concanavalin slides for 30 min. Fixation was performed for 15 min in 4% paraformaldehyde, followed by washing in PBS. Nuclei were permeabilized in PBS with 0.3% Triton, and blocked in: PBS, 0.3% Triton, 2% BSA. Primary antibody incubation was performed over-night in PBS-T/BSA and appropriate secondary antibody incubation for 1 h. Slides were mounted in Mowiol (Calbiochem-Novabiochem) containing 0.2 ng/ml DAPI (Sigma), visualized in a Leica TCS SPE confocal microscope, and analyzed using FIJI software.

### ChIP experiments

For preparing chromatin for ChIP, crosslinking of DREX-incubated and control nuclei was performed in 1.8% formaldehyde for 10 min at room temperature. Glycin (125 mM) was added to stop the reaction. Nuclei were washed with PBS, Wash Buffer A (10 mM HEPES pH 7.9, 10 mM EDTA, 0.5 mM EGTA and 0.25% Triton X-100) and Wash Buffer B (10 mM HEPES pH 7.9, 100 mM NaCl, 1 mM EDTA, 0.5 mM EGTA, 0.01% Triton X-100). The pellet was then resuspended in TE (10 mM Tris-HCl pH 8, 1 mM EDTA) and 1% SDS was added, followed by centrifugation at 3300 g/10 min, at 4 °C. TE wash was performed. Then TE buffer with 0.1% SDS and 1 mM PMSF was added. Sonication was performed in 15-ml tubes in Bioruptor sonicator where 26 sonication cycles of 30 s ON/30 s OFF were performed at high intensity. To check the size of the sonicated DNA part of the sample was checked on an agarose gel after de-crosslinking. In continuation, 1% Triton X-100, 0.1% Deoxycholate and 140 mM NaCl were added. Preclearing of chromatin samples was performed with Protein A sepharose for 1 h, followed by addition of antibody and overnight incubation at 4 °C. Incubation was continued for additional 4 h upon Protein A sepharose addition. Five washes with RIPA buffer (140 mM NaCl, 10 mM Tris-HCl pH 8, 1 mM EDTA, 1% Triton X-100, 0.1% SDS, 0.1% Deoxycholate), one wash with LiCl ChIP buffer (250 mM LiCl, 10 mM Tris-HCl pH 8, 1 mM EDTA, 0.5% NP-40 and 0.5% Deoxycholate) and two washes with TE buffer were performed. Samples were RNAse-treated. De-crosslinking was performed overnight at 65 °C upon addition of 0.1 M NaHCO_3_ and 1% SDS. In continuation, samples were treated with Proteinase K and DNA extraction with Phenol–Chloroform followed by EtOH precipitation was performed.

For ChIP-qPCR, triplicates were subjected to real-time PCR using SYBR Green I Master Mix and LightCycler® 480 Instrument (Roche). Percentages of immunoprecipitated material were calculated by the ΔΔCt method. Primers used in these experiments are listed in Supplementary Table S1.

### Data availability

All data generated or analysed during this study are included in this published article (and its Supplementary Information files).

## Electronic supplementary material

Supplementary Information
