# Supplementary material for: Chromatin remodeling in Drosophila preblastodermic embryo extract
Source: Sci Rep. 2018 Jul 19;8:10927. doi: 10.1038/s41598-018-29129-8 (PMC6053431; doi:10.1038/s41598-018-29129-8)
Supplement: Supplementary file 1 — Supplementary Information [file 41598_2018_29129_MOESM1_ESM.docx]

**Chromatin remodeling in *Drosophila* preblastodermic embryo extract**

Eva Šatović, Jofre Font-Mateu, Albert Carbonell, Miguel Beato and Fernando Azorín

**Supplementary Figures and Tables**

**Supplementary Figure S1. Incubation in DREX induces binding of dBigH1 to somatic S2 nuclei chromatin.** (**a**) IF of somatic S2 nuclei with αdBigH1 antibodies (green) after incubation in DREX for the indicated times. DNA was stained with DAPI (blue). (**b**) As in (**a**) but for αdH1 (red) and αdBigH1 antibodies (green). Scale bars are 20 µm. Quantitative analysis is sown in the right, where the correlation between mean grey αH1 and αdBigH1 immunofluorescence is presented for individual nuclei (N=140) upon 1h incubation in DREX.

**Supplementary Figure S2.** **Increased histone acetylation induced by DREX does not depend on dBigH1 binding. (a)** WB analyses of the amount of dBigH1 bound to chromatin after incubation of S2 nuclei in DREX for the indicated times in the presence or not of αdBigH1 antibodies. Quantitative analysis of the results is shown in the bottom. Data are presented as mean ± SD. (N=2; two-tailed T-test p-values **<0.01, ***<0.001) (**b)** As in **a** but for global levels of H3Ac. Data are presented as mean ± SD. (N=2; two-tailed T-test p-values >0.05). Full-size films or scans of the blots are presented in Supplementary Figures S12 and S13 (biological replicates).

**Supplementary Figure S3. The effects of incubation in DREX on global levels of active RNApol II forms and H3K27me3.** WB analyses of the global levels of the promoter-proximal RNApol II (IIo^ser5^) (**a**), the elongating RNApol II (IIo^ser2^) (**b**) and H3K27me3 (**c**). Data are presented as normalized mean ± SD. (N=3 (for IIo^ser5^) and 2 (for H3K27me3); two-tailed T-test p-values *<0.05). Full-size films or scans of the blots are presented in Supplementary Figures S14 and S15 (biological replicates).

**Supplementary Figure S4. Incubation in DREX induces disassembly of HP1a foci.** IF with αHP1a (red) and αdBigH1 (green) of somatic S2 nuclei after incubation in DREX (**b**) or in control (**a**) conditions for the indicated times. DNA was stained with DAPI (blue). Scale bars are 20 µm.

**Supplementary Figure S5. Incubation in DREX induces disassembly of H3K9me3 foci.** (**a**) IF with αH3K9me3 (red) of somatic S2 nuclei after incubation in DREX or in control conditions for the indicated times, and prior to incubation. DNA was stained with DAPI (blue). Scale bars are 20 µm. (**b**) Enlarged images of nuclei immunostained with αH3K9me3 (red) after incubation in DREX (right) or in control (left) conditions for 1h. Scale bars are 3 µm. In the bottom, the proportions of nuclei showing extruded H3K9me3 foci or not are presented after incubation in DREX or in control conditions for 2h. (N= 447 for DREX; N= 355 for control)

**Supplementary Table S1**. Primers used in these experiments

| **ChIP-qPCR PRIMERS** | **forward** | **reverse** |
| --- | --- | --- |
| \|  \| α-Tubulin \| \| --- \| --- \| | ACTACGGCAAGAAGTCCAAG | GTGGTCAGGATGGAGTTGTA |
| RpL23 | GACAACACCGGAGCCAAGAACC | GTTTGCGCTGCCGAATAACCAC |
| ATP synthase | ATGTGTACTCGGAGGTGAAG | CATTGGTGGTGATCCAGTTG |
| Hexokinase A | TTGCCACTCGGTTTCACCTT | AACATCCTCGTTGACGACGC |
| α spectrin | AACACGCTGAAGGAGAAGTC | CCATAATCACTGCCAGTTGC |
| zipper | TGCGAGAAGATGATTCAGGC | CTTGAAATCACGCTCCTCCT |
| vinculin | CAGAATGCCCTGGTCAATCG | GTTGAAGTTCTGCTCCCGTC |
| bottleneck | TCCACCAACAACCGCTTCAT | TCACGGAACCACTGAGACTG |
| brother | AGGTTCGCTACACAGGATAC | GTGGTTGGCGTTGAAGTAGA |
| Blastoderm-specific gene 25A | AGTTCGGAGCCTTTTAACGC | AGTCCTCATCGTCATCGTCC |
| CG14317 | ATGTTGCCCAATCTCAAGCG | AGATATCCTGAATCCAGCGG |
| bottleneck TSS | TATAAGGTCTGCGTTCCTGG | GCTTGTAGTTGTAGAACTGGAAG |
| brother TSS | AGGCGGTCAATTTTAACATTGATC | ATCATTCCATTCATGGCGGC |
| Blastoderm-specific gene 25A TSS | AATCAGTATTTGGAAGAAACTACCT | TCATTGTGCTGCTTGTTGTC |
| CG14317 TSS | CAAGAGCACATTCAACCAACAG | GAGCGTCCTTGTAGAATGC |
| Ftz TSS | GGCTCTCTGATTTTGCTATATATGC | TAGTGGCTCTGGCTGTTTGT |
| Hairy | CGCCGATCCCAAGATTGTG | AGCGGCTAACCTCGTTCAC |
| Eve | AACTCCTTGAACGGCAGCC | CAGCTGGTCACGGGTGAAG |
| Ftz | GTCAGACGTACACCCGCTA | CGGGTGATGTATCTATTGA |
| Tum | GGAACACCGACGACGAAG | TACGCAGTGCACAATCAGT |
| Actin | GCGCGGTTACTCTTTCACCA | ATGTCACGGACGATTTCACG |
| Act5C_TSS | AAGCGGGCTTTATAAAACGG | GGTTTGGTGTCTCTGGATTA |
| RT96500 | GAATCGAAAACTCACGTAGCA | TGGTTGCTACTTTACTTGCGC |
| Gypsy ORF | CCTCAGAGCTGTGGTCTTCC | CAGATGGCAGGTCTTTTGGT |
| TR 2 | AAACACGTCTCCACCCGAAG | CTATTCTAACATTCGGCATTCCAC |
| TART ORF | CCAATGCAACCAAAGCATTA | TATGTGTGGGAGGGAGAAGC |
| Het-AORF | AAACGACGATCTGGACTGCT | CGGAAAAATGCTGGGAGTTA |
| ACCORD ORF | CCAACAGCAACAACATGGAC | AAAAGCCAAAATGTCGGTTG |
| Diver2 LTR | CGCCAAAACTGTGCAGTAGA | CAGATAAATGCGTGCGAGAA |
| DOC ORF | CGCTGTGCCAGCTGTAAATA | ATTGTTGTTGCAAACGGTCA |
| GATE LTR | CCGCTCTTCACCTCAGAGTC | CCGGGCGTATGTTTATTCAG |
| GYPSY LTR | GGCTCATTGCCGTTAAACAT | GGCGATAGCGATTTGATTGT |
| Het-A UTR | TTCGCTTGCCAAAGACTCTC | GCTTTTCTTTGCAGCCTGAG |
| Invader4 LTR | AGATGACAATGTGGCACACG | GATCGACGTCAGCAGTCAAA |
| Invader4 ORF | GCTTACGCCTTCAAGAAACG | CAAAAATGGCACATGGTCTG |
| Pogo ORF | TACATTTGGTTCGGACAGCA | ACGTGCCGGTCAAGAATTAC |
| satDNAIII 359 bp | TATTCTTACATCTATGTGACC | GTTTTGAGCAGCTAATTACC |
| satDNA 260 bp | ATGAAACTGTGTTCAACAAT | TGGAAATTTAATTACGAGCT |
| DCTN3_TSS | ATCGATGTCCAACGAAAAG | TCCGCATAAGGGTTAATTCG |
| Ibf2_TSS | TTGGAAGAAACTGGGCTTTC | TCAGCCTGTCTATGCTGGTG |
| Cnx99A_TSS | CAGTGTGACCAGTTGTCCTT | GATCTGGCACGACGAACTAT |
| Pgm_TSS | GACGCACCCCCAATACTAGA | CCTCCCACTGATAAGCCAAA |
| CG4038_TSS | TGCCTAGTGTGACCGTATAAT | CGCGTTCCGATCAACCAAA |

**Full-size films or scans of the blots presented in main text Figures, Supplementary Figures and the corresponding biological replicates**

**
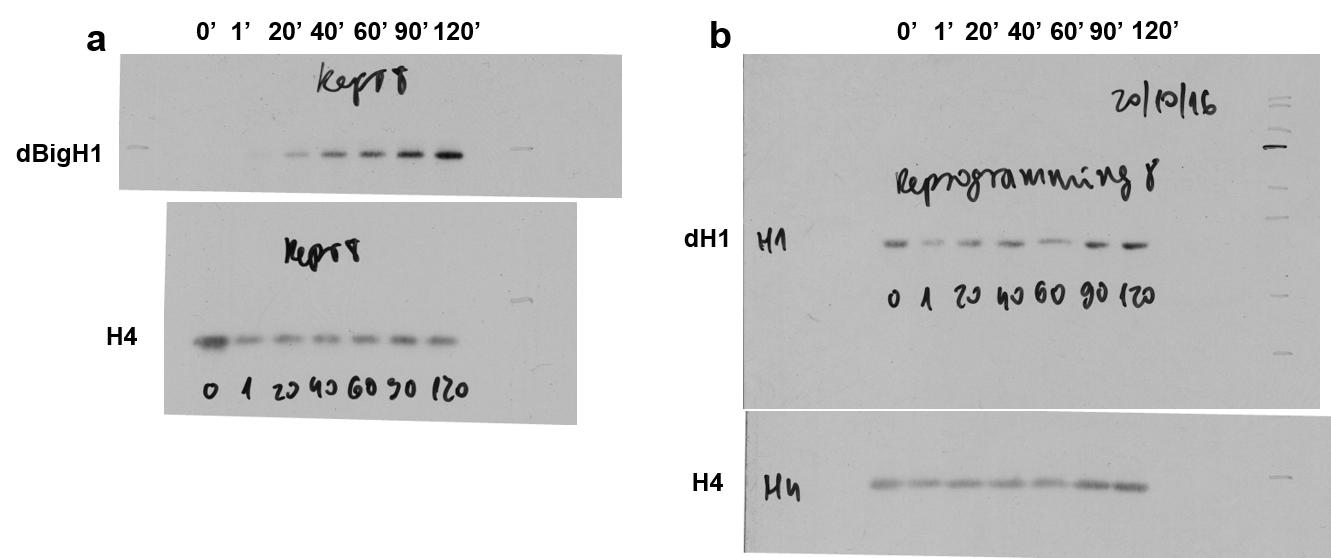
**

**Supplementary Figure S6: Full-size films of the blots presented in Figure 1a and 1c. (a)** dBigH1 (up) and H4 (down) (**b**) H1 (up) and H4 (down)

**
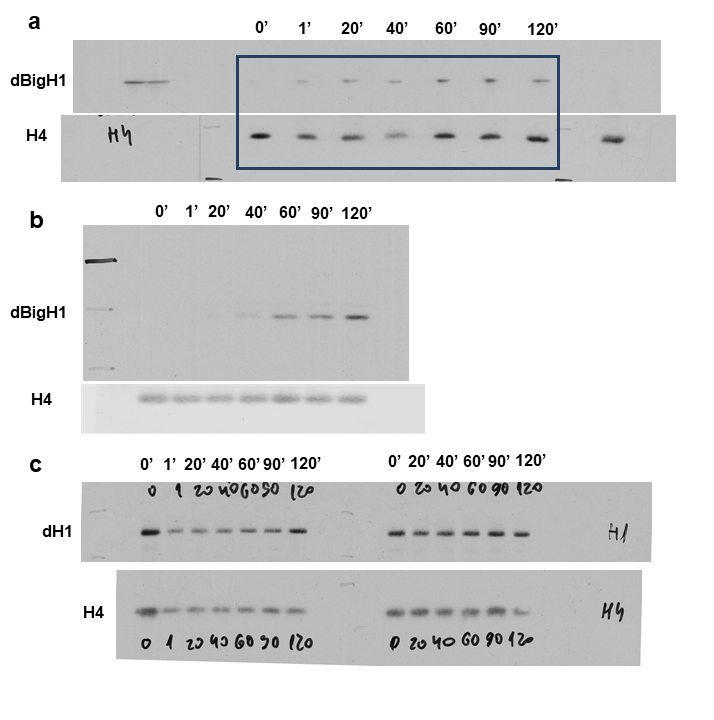
**

**Supplementary Figure S7: Full-size films of the biological replicates from the wb experiments shown in Figure 1a and 1c.** (**a**) dBigH1 (up) and H4 (down) (**b**) dBigH1 (up) and H4 (down) (**c**) H1 (up) and H4 (down)

**
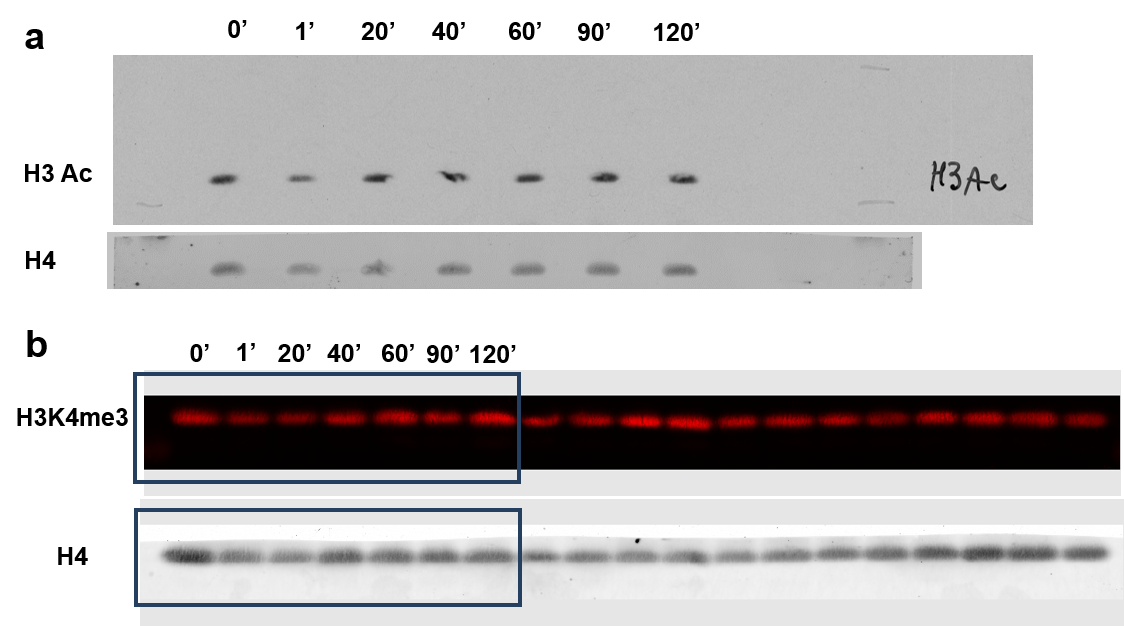
**

**Supplementary Figure S8: Full-size films or scans of the blots presented in Figure 2a and 2c.** (**a**) H3 Ac (up) H4 (down) (**b**) H3K4me3 (up) H4 (down)

**
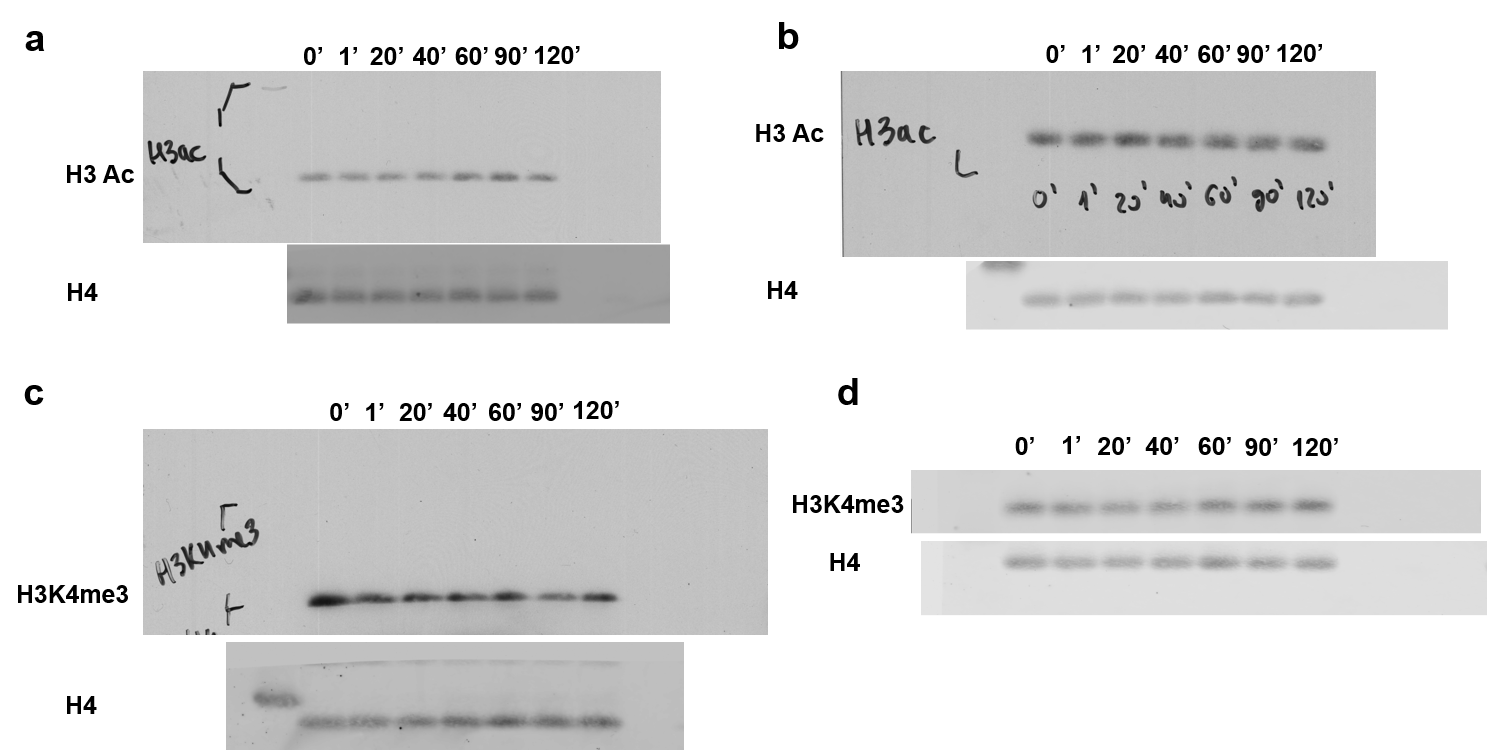
**

**Supplementary Figure S9: Full-size films or scans of the biological replicates of the wb experiments shown in Figure 2a and 2c.** (**a**) H3 Ac (up) and H4 (down) (**b**) H3 Ac (up) and H4 (down) (**c**) H3K4me3 (up) and H4 (down) (**d**) H3K4me3 (up) and H4 (down).

**
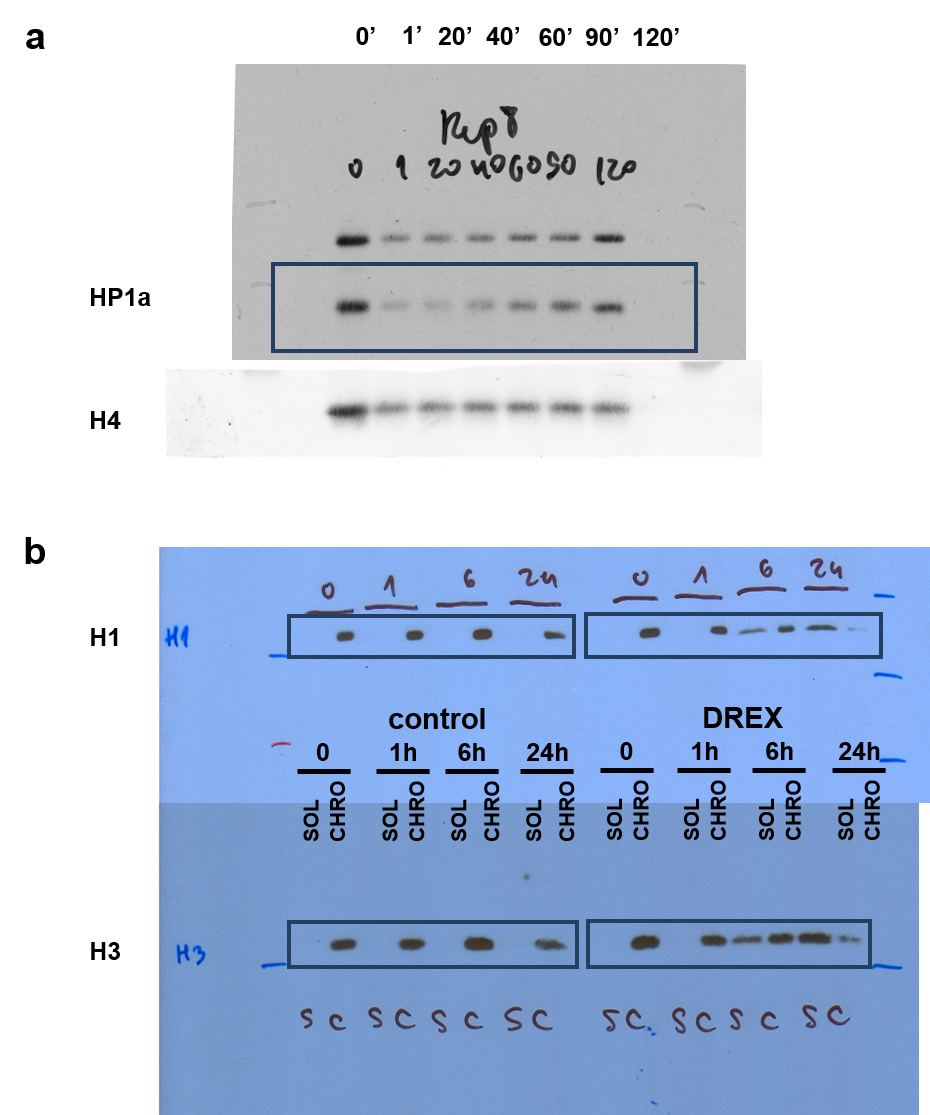
**

**Supplementary Figure S10: Full-size films or scans of the blots presented in Figure 3c and d.** (**a**) HP1a (up, in rectangular) H4 (down) (**b**) up H1, left – DREX, right – control; down H3: left – DREX, right – control


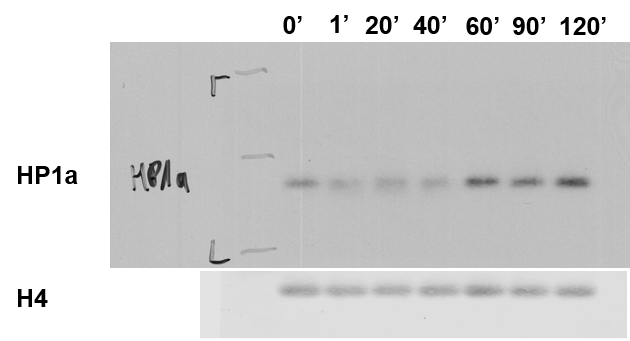


**Supplementary Figure S11: Full-size films or scans of the biological replicate of the experiment shown in Figure 3c.** HP1a (up) and H4 (down)

**
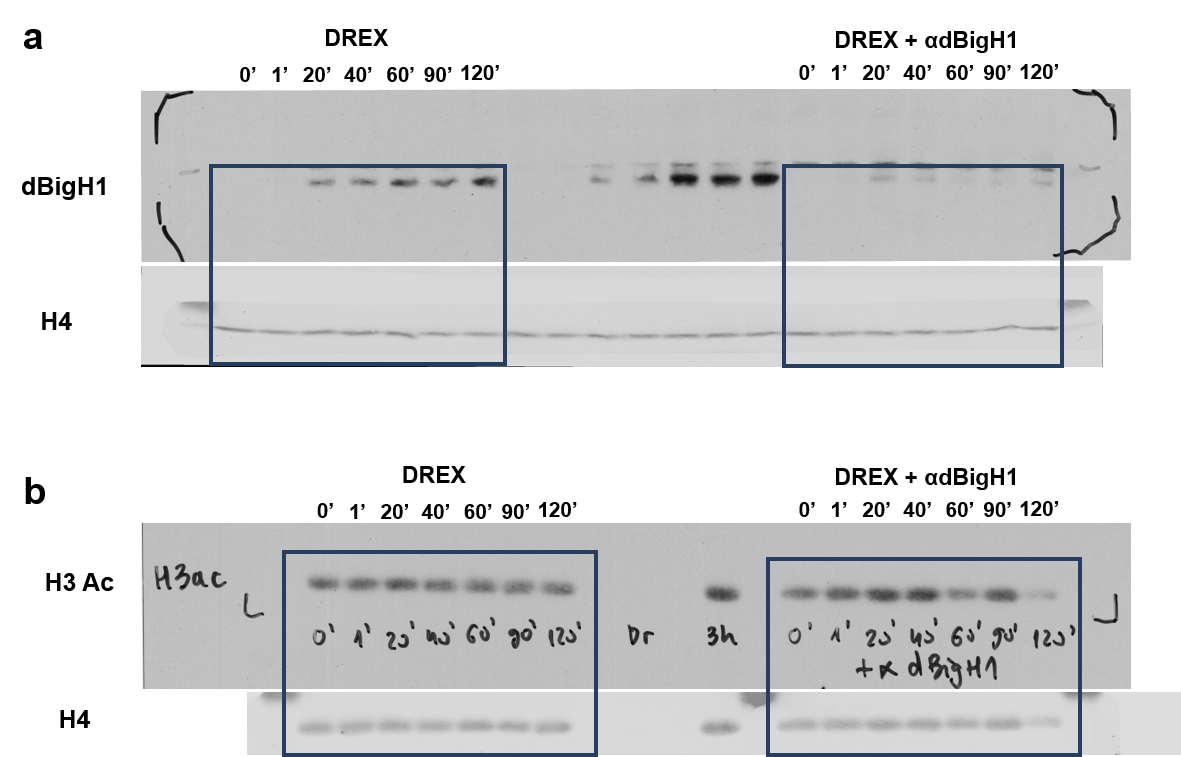
**

**Supplementary Figure S12: Full-size films or scans of the blots presented in Supplementary Figure S2a and b.** (**a**) up dBigH1: left DREX, right DREX + αdBigH1; down H4: left DREX, right DREX + αdBigH1 (**b**) up H3 Ac: left DREX, right DREX + αdBigH1; down H4: left DREX, right DREX + αdBigH1

**
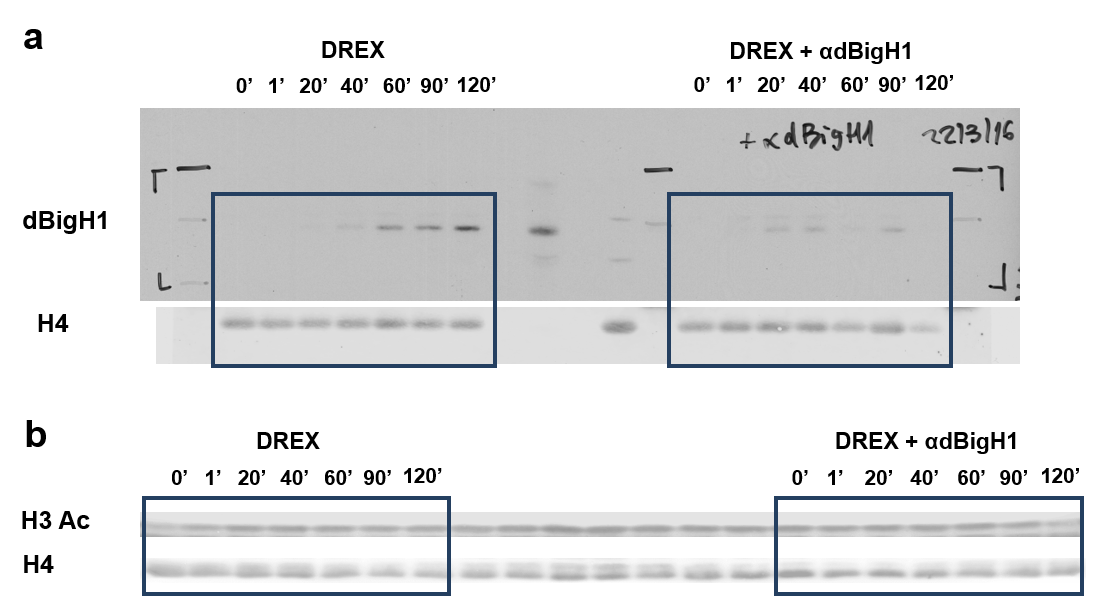
**

**Supplementary Figure S13: Full-size films or scans of the biological replicates of the experiments shown in Figure S2a and b.** (**a**) up dBigH1: left DREX, right DREX + αdBigH1; down H4: left DREX, right DREX + αdBigH1 (**b**) up H3Ac: left DREX, right DREX + αdBigH1; down H4: left DREX, right DREX + αdBigH1 (down)


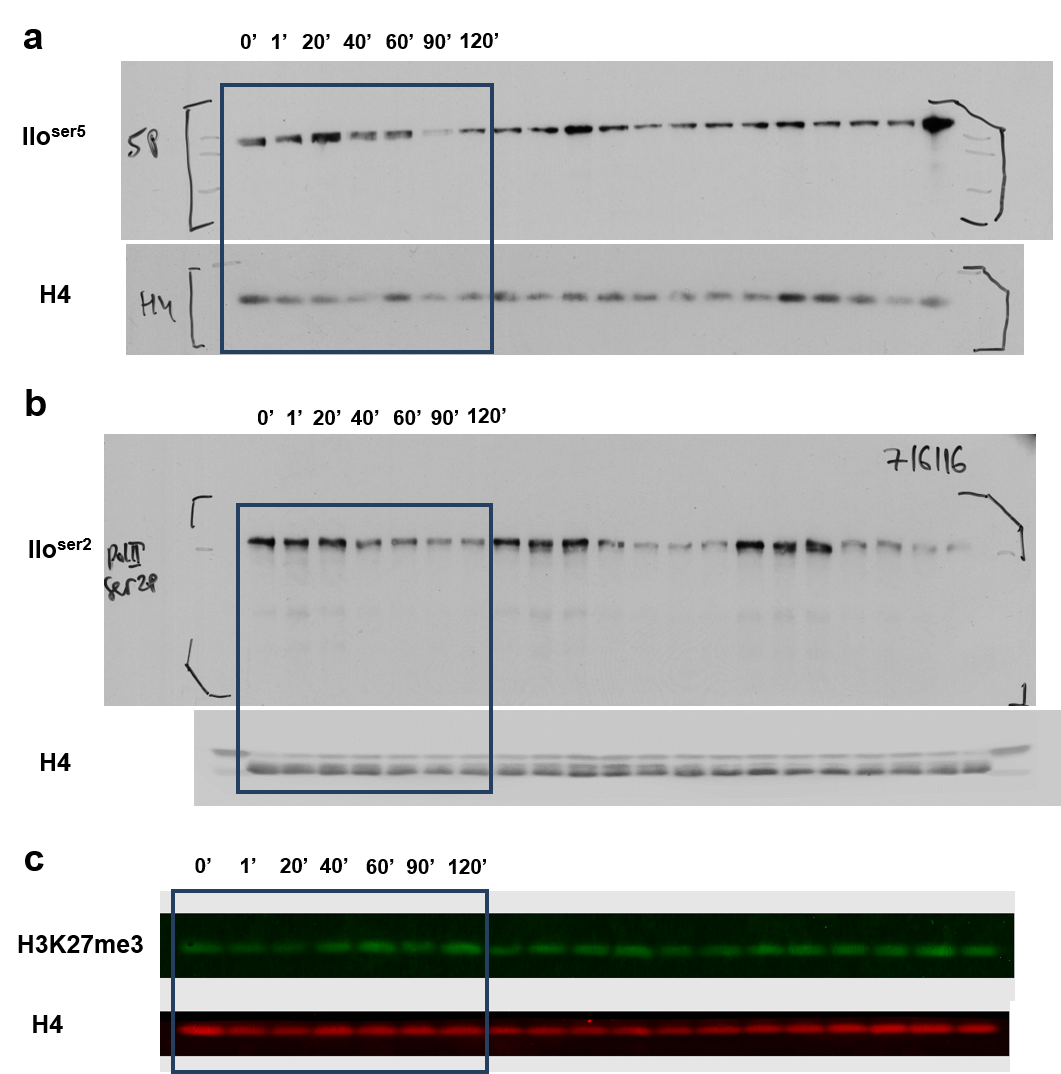


**Supplementary Figure S14: Full-size films or scans of the blots presented in Supplementary Figure S3.** (**a**) RNApol II (IIo^ser5^) (up) and H4 (down) (**b**) RNApol II (IIo^ser2^) (up) and H4 (down) (**c**) H3K27me3 (up) and H4 (down)

**
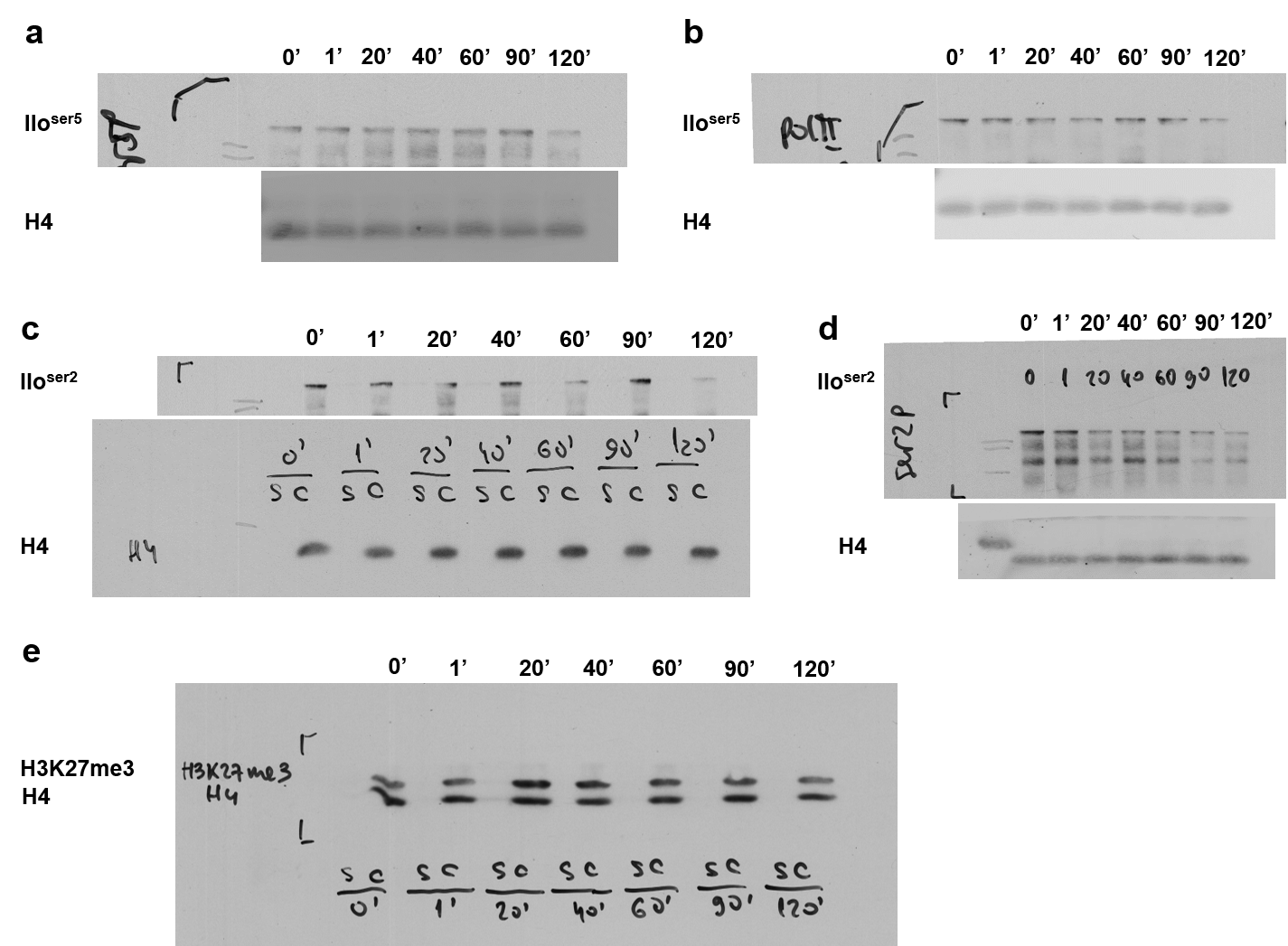
**

**Supplementary Figure S15: Full-size films or scans of the biological replicates of the experiments shown in Supplementary Figure S3.** (**a**) IIo^ser5^ (up) and H4 (down) (**b**) IIo^ser5^ (up) and H4 (down) (**c**) IIo^ser2^ (up) and H4 (down), (**d**) IIo^ser2^ (up) and H4 (down), (**e**) H3K27me3 (up) and H4 (down).
